# Creating Tunable Micro-Optical Components via Photopolymerization 3D Printing Combined with Polymer-Dispersed Liquid Crystals

**DOI:** 10.3390/mi16010026

**Published:** 2024-12-28

**Authors:** Sheng-Yuan Zhang, Hsi-Fu Shih, Chuen-Lin Tien, Han-Yen Tu

**Affiliations:** 1 Department of Mechanical Engineering, National Chung Hsing University, Taichung 40227, Taiwan; l1502wwww23@gmail.com; 2 Department of Electrical Engineering, Feng Chia University, Taichung 40724, Taiwan; cltien@fcu.edu.tw; 3 Department of Electrical Engineering, Chinese Culture University, Taipei 11114, Taiwan; dhy@ulive.pccu.edu.tw

**Keywords:** 3D printing, photopolymerization, polymer-dispersed liquid crystal (PDLC), diffraction grating, Fresnel zone plate

## Abstract

Based on additive manufacturing via photopolymerization, this study combines polymer-dispersed liquid crystal (PDLC) technology with 3D printing technology to produce tunable micro-optical components with switchable diffraction or focusing characteristics. The diffraction grating and Fresnel zone plate are the research targets. Their structures are designed and simulated to achieve expected optical functions. A liquid crystal display (LCD) 3D printer is used to produce structures on transparent conductive substrates. The printed structures are filled with PDLCs and covered with transparent conductive substrates to achieve tunable functions. The proposed configurations are implemented and verified. The experimental results show that the diffraction efficiency of the 0th order increases from 15% to 50% for the diffraction grating and the focusing spot intensity decreases from 74% to 12% after the application of an electric field. These results demonstrate the feasibility of the proposed tunable optical component configurations.

## 1. Introduction

Micro-optical components are elements indispensable for many technological products, especially thin, light, and portable devices or optical, mechanical, and electrical systems. Therefore, they play very important roles. In general, most micro-optical components are manufactured via micro-fabrication methods, including lithography, etching, and lithographie–galvanoformung–abformung (LIGA) processes, and with complex and expensive equipment [1]. This makes the design and development processes of optical components lengthy and costly. Recently, some studies on the use of 3D printing for optical or micro-optical component manufacturing have been reported. For example, Willis et al. presented 3D-printed embedded optical elements, such as light pipes for illumination, sensors, or the display of customized interactive device applications [2]. Maillard et al. designed and fabricated a freeform optical sensor using 3D printing by integrating optical elements like mirrors and lenses into a simple printed element for metrology applications [3]. Cook et al. presented a 3D-printed structured preform for the structured optical fiber drawing of waveguide fabrication applications [4]. Similarly, Iezzi et al. used 3D printing technology for plastic fiber fabrication with long-period grating structures for filter or sensor applications [5]. Juodkazis used two-photon absorption 3D laser microprinting technology to produce micro-optical compound lenses that can be used on a CCD image sensor surface or directly on the tip of an optical fiber for endoscopy applications [6]. Siegle et al. further utilized two-photon grayscale lithography 3D printing to create complex aspherical singlet and doublet micro-optical step-free lenses [7]. Kotz et al. created transparent fused silica glass components using a stereolithography 3D printer to print and convert photocurable silica nanocomposites into high-quality fused silica glass via heat treatment for macro- and microstructures [8]. Hong et al. developed a two-photon 3D printing process using a liquid and solvent-free silica precursor for glass micro-optics [9]. Aslani et al. presented different methods to fabricate colored microlenses by adding pigments to polymers or dyeing the printed structures in a post-printing process [10].

Three-dimensional printing allows for rapid prototyping, environmental protection, energy savings, material savings, lower equipment costs, etc. Common 3D printing technologies can generally be divided into four categories: power bed fusion (PBF), sheet lamination (SL), material extrusion (ME), and vat photopolymerization (VP). VP uses ultraviolet (UV)-sensitive polymer material combined with an optical projection system to solidify 3D objects, and its advantages include the use of cheap materials and simple equipment. Three types of VP can be classified based on the employed optical projection method: stereolithography lithography apparatus (SLA), digital light processing (DLP), and liquid crystal display (LCD). All of them can provide high-quality and detailed printing with better structural accuracy and higher resolution compared with other 3D printing methods. In addition, VP is capable of printing transparent objects by using transparent photopolymers as the printing materials. Therefore, it is a good candidate for 3D printing plastic optical components and is widely adopted in research and industry applications [11,12,13,14,15].

With the rapid changes in technological products, single-function micro-optical components have gradually failed to meet the necessary requirements. Therefore, tunable devices with multiple functions are beginning to be urgently needed; these include focusing lenses with adjustable focal lengths, gratings with adjustable diffraction efficiencies, beam splitters that can switch the light path, modulators that can scan the laser beam, etc. Generally, these components cannot be created through a single traditional processing method but require many manufacturing methods associated with complex process technologies. Therefore, some studies have proposed a new concept of promoting 3D printing to 4D printing to add functions to originally fixed printed structures and create tunable devices with simpler manufacturing processes [16]. For example, Jeong et al. discussed 3D and 4D printing for optics and metaphotonics [17]. Ali et al. proposed 4D-printed thermochromic Fresnel lenses for sensing applications [18]. After the creation of these lenses, that team extended 4D printing to 5D printing [19].

Based on the above description, we proposed an innovative architecture that combined photopolymerization 3D printing with polymer-dispersed liquid crystal (PDLC) technology [20,21,22,23,24] to produce tunable micro-optical components. Three-dimensional printing was adopted to create the basic frame of the micro-optical component structure. The PDLC mixture was used to fill in gaps in the structure, and the tunable effect was achieved by applying an external electric field. This design specifically targeted optical diffraction grating and Fresnel zone plate applications.

## 2. Design and Manufacturing

**Figure 1:**
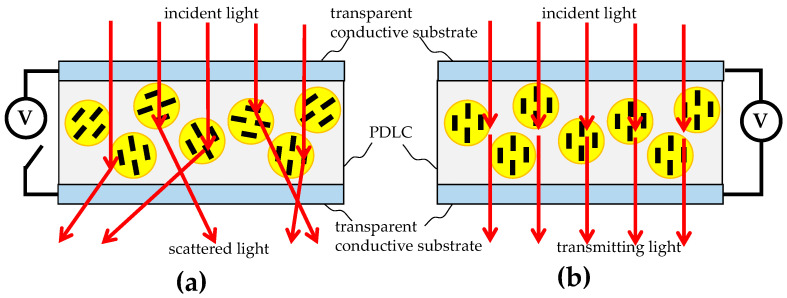
Schematic diagrams of the operating mechanism of PDLCs, where (**a**) shows that the incident light is scattered by randomly oriented LC droplets when an electric field is not applied and (**b**) shows that the incident light is completely transmitted through the PDLCs when an electric field is applied.

Generally, liquid crystal (LC) devices require an alignment layer to be coated on the upper and lower transparent conductive substrates in order for the liquid crystal molecules to be properly aligned. However, the steps for such a process are slightly cumbersome, so PDLC technology was developed, which uses LCs and polymers to form a condensed system. LC droplets are randomly dispersed into the polymer. Due to the size and the internal director of the LC droplets matching the index of refraction of the polymer, the PDLCs can be driven by the electric field of the upper and lower transparent conductive substrates, such as indium tin oxide (ITO) substrates, without the need for alignment layers. They can provide two states of light scattering and penetration. Figure 1 shows schematic diagrams of the mechanism by which PDLCs operate.

As shown in Figure 1a, when an electric field is not applied, the incident light is scattered by randomly oriented LC droplets, while as shown in Figure 1b, when an applied electric field is applied, the incident light is completely transmitted through the PDLCs. Since no alignment is required, the manufacturing process of PDLCs is relatively simple and suitable for mass production.

### 2.1. Design Concept

**Figure 2:**
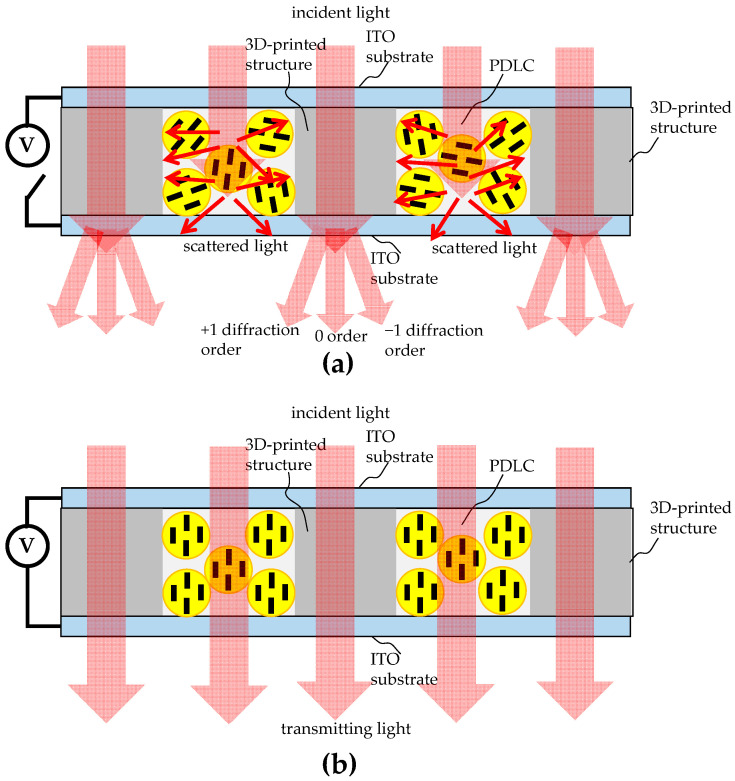
Schematic diagrams of the design concept, where (**a**) shows that when no electric field is applied to PDLCs, light is scattered by the PDLCs, and the 3D-printed structure diffracts the incident beam into several diffraction orders, and (**b**) shows that light is not scattered by the PDLCs due to an external electric field and the incident beam nearly completely penetrates through the component.

The main advantage of 3D printing is that it is not limited by arbitrary physical structures and can be directly produced layer by layer according to the target component drawn by computer-aided design (CAD) software (SolidWorks 2020). If 3D printing is used to produce optical components, the quality of the refractive optical elements (ROEs) struggles to reach an optical mirror grade due to the poor surface flatness of the stacked layers. Therefore, 3D printing is more suitable for the production of diffractive optical elements (DOEs), especially for DOEs that are structured in binary or multiple levels (also known as Kinoform elements [12]). With this in mind, the adoption of a binary DOE configuration as the base architecture formed by photopolymerization 3D printing and then the combination of the DOE configuration with PDLCs to create tunable micro-optical components with switchable diffraction efficiency or focusing characteristics are feasible. Figure 2 shows a schematic diagram of the design concept. The PDLCs were used to fill the gaps between adjacent 3D-printed structures. Referring to Figure 2a, no electric field was applied to the PDLCs, and the directors of the LC droplets were randomly distributed. Light was thus scattered by the PDLCs. This configuration made the device act like periodic amplitude grating and diffracted the incident beam into several diffraction orders. Once an external electric field was applied to the PDLCs, the PDLCs did not scatter any light, as shown in Figure 2b. Due to the index between the PDLCs and 3D-printed polymers matching, the periodic grating structure nearly disappeared, the incident beam nearly penetrated the component, and no diffraction occurred.

### 2.2. Device Design Configuration

In order to verify the above idea, two types of tunable micro-optical components were proposed, one for the diffraction grating and the other for the Fresnel zone plate. They were configured as follows.

#### 2.2.1. Diffraction Grating

**Figure 3:**
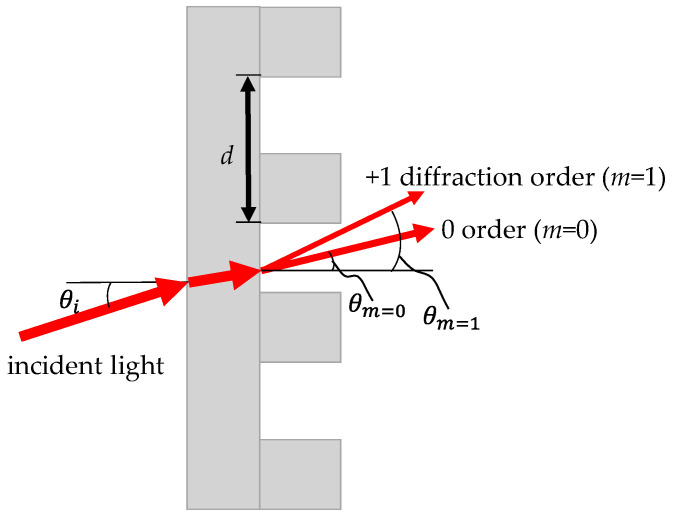
Schematic diagram of the relationship between the diffraction angles and grating period.

Referring to Figure 3, the relationship between diffraction angles and grating period can be calculated from the following grating formula, Equation (1) [25].

(1) $$d\left(sin\theta_{m}-sin\theta_{i}\right)=m\lambda$$

where d is the grating period, m is the diffraction order, λ is the wavelength, $\theta_{i}$ is the incident angle, and $\theta_{m}$ is the diffraction angle of the mth order. The diffraction efficiency can be calculated based on the far-field diffraction of the Fourier transform of the grating profile.

**Figure 4:**
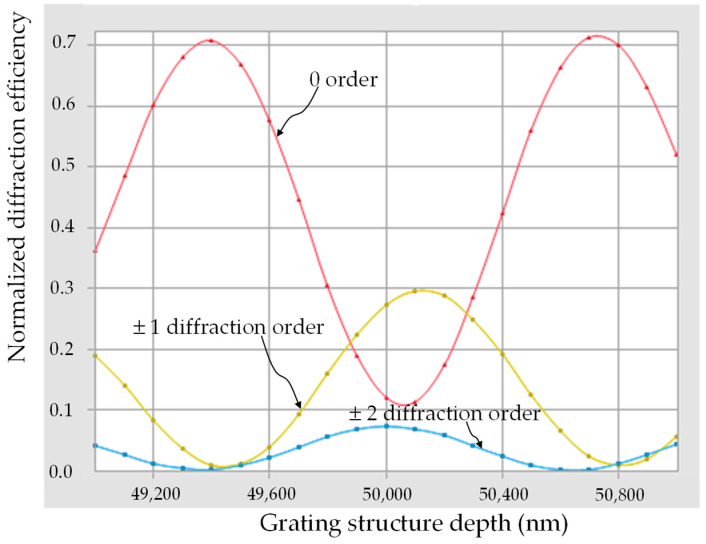
Simulated diffraction efficiencies for primary orders corresponding to grating structure depth.

For convenience, the rigorous diffraction grating analysis tool Gsolver (V5.2) [26] was adopted to calculate the diffraction angles and efficiencies of a periodic grating structure for each diffraction order. The parameters for simulation are listed in Table 1, and the simulated diffraction efficiencies for primary orders corresponding to grating structure depth are shown in Figure 4. A CAD file of the grating structure was generated using these parameters and uploaded to the LCD 3D printer. The 3D grating structure was then printed layer by layer onto an ITO substrate. After printing was completed, the liquid PDLC mixture was added to cover the top of the structure and exposed to UV light so that the LC droplets could be fixed in place via polymerization. Finally, the upper ITO substrate was attached to finish producing the component. Due to the limitation of the printing resolution of the LCD 3D printer, a grating period of 500 μm and a duty cycle of 25% were chosen for the design. When no voltage was applied to the upper and lower ITO substrates, the PDLC mixture of the device scattered the incident beam, and the 3D-printed part allowed the incident beam to penetrate the substrates, forming a periodic amplitude grating, which caused the incident beam to generate several diffraction orders—the expected optical effect of the element. When a voltage was applied between the upper and lower ITO substrates, the incident light beam completely penetrated both the PDLC mixture and the 3D-printed part of the device. Since both are polymer materials, their indices of refraction are similar and match mutually, and nearly no phase differences exist within the structures. The grating structure was not obvious and did not cause diffraction of the incident beam, showing complete penetration.

#### 2.2.2. Fresnel Zone Plate

**Figure 5:**
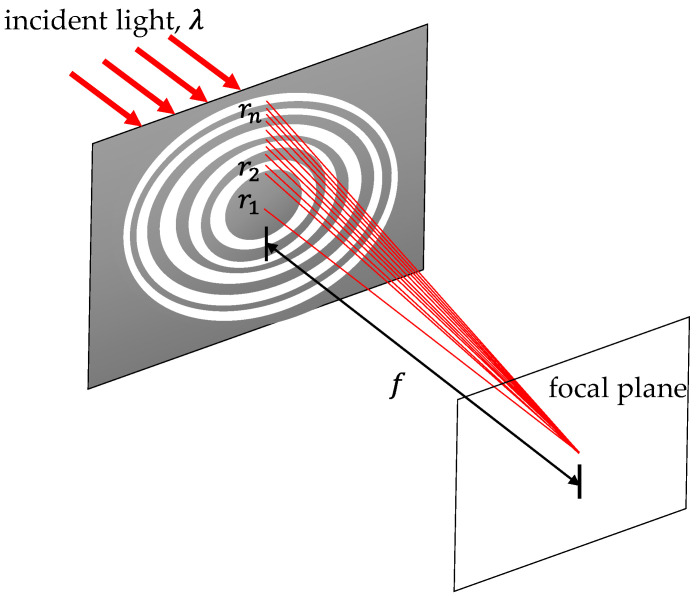
Optical diagram of a Fresnel zone plate.

**Figure 6:**
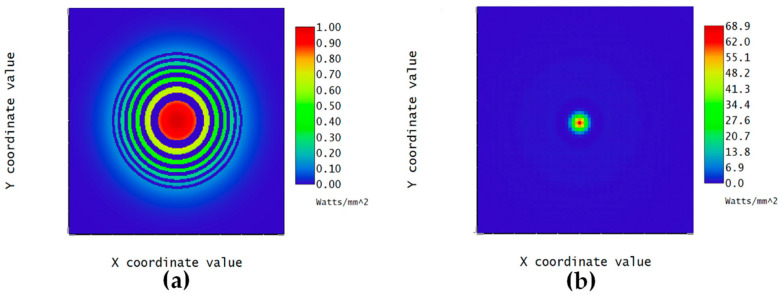
Simulated intensity distributions on (**a**) the plane just after the zone plate and (**b**) the focal plane.

Figure 5 shows the optical diagram of a Fresnel zone plate. The design flow is similar to that described above for the diffraction grating. In order to create a device with focusing capabilities, the radii of zones was calculated according to Equation (2) [25].

(2) $$r_{n}≅\sqrt{nf\lambda }$$

where $r_{n}$ is the radius of the *n*th zone and f is the primary focal length of the Fresnel zone plate. Table 2 lists the calculated radii of ten zones for the targeted zone plate with a focal length of 8 m operated under a wavelength of 632.8 nm. With these values, the focal length and focusing characteristics of the zone plate were simulated using the optical design software Zemax 2010 [27]. Figure 6 shows the simulated intensity distributions on the plane just after the zone plate and the focal plane. The distribution confirms the focusing capability of the zone plate design. Moreover, the CAD drawing for the 3D structure of the zone plate was obtained and uploaded to the LCD 3D printer. The subsequent steps, including filling the PDLCs and applying a voltage, were the same as those carried out for the diffraction grating. When no voltage was applied between the upper and lower ITO substrates, the PDLC mixture of the device scattered the incident beam and the 3D-printed part allowed the incident beam to penetrate the substrates, forming a Fresnel zone plate of amplitude type as a whole, which caused the parallel incident beam to converge at a spot, creating a focusing effect for the element. When a voltage was applied, the structure inside the plate was not obvious and did not cause any focusing of the incident beam, showing complete penetration.

### 2.3. Manufacturing Results

**Figure 7:**
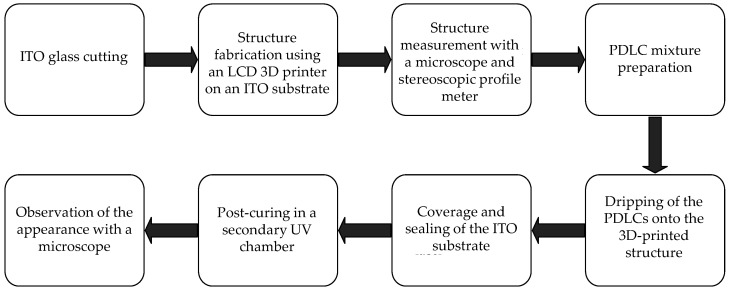
The device manufacturing process.

**Figure 8:**
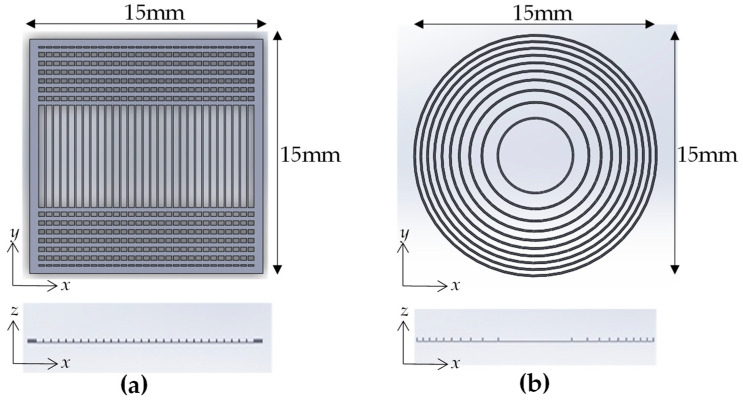
CAD models for (**a**) the diffraction grating and (**b**) the zone plate.

Figure 7 shows the complete device manufacturing process. First, the ITO substrate with its conducting surface was placed onto the moving platform of the LCD photopolymerization 3D printer (brand name: Phrozen mini 8k [28]). The preprocessing software Chitubox Basic [29] was used to slice the CAD into layers before uploading it to the printer. Table 3 lists the main software settings and the associated parameters. Then, the printer illuminated the transparent UV-curable resin (brand name: Aqua-Clear, index of refraction: 1.512) layer by layer using 405 nm light, exposing each layer for 13 s and displaying the pattern from the uploaded sliced CAD model, as shown in Figure 8. This process creates a printed 3D structure for a diffraction grating or a Fresnel zone plate on the ITO substrate.

**Figure 9:**
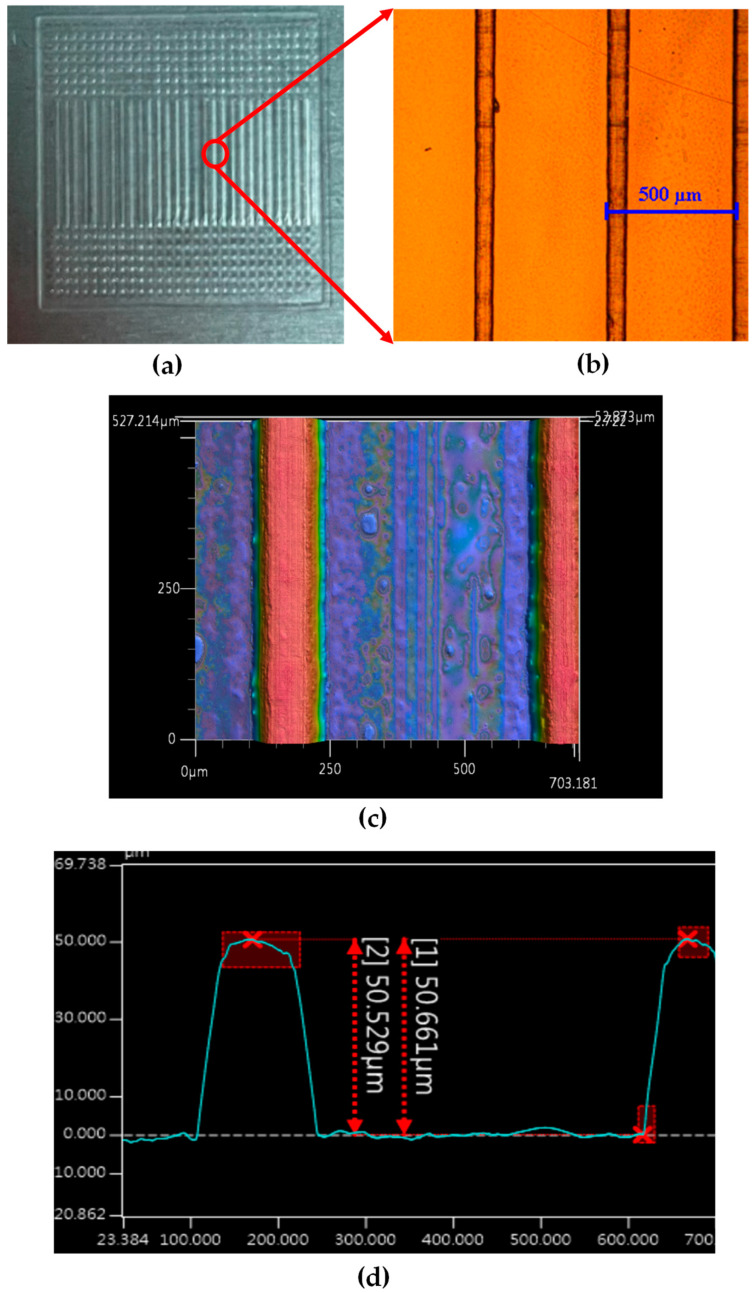
Measurements of the 3D-printed diffraction grating structure: (**a**) device, (**b**) microscopic view, (**c**) stereoscopic profile image, and (**d**) structure depth measured with the stereoscopic profile meter.

**Figure 10:**
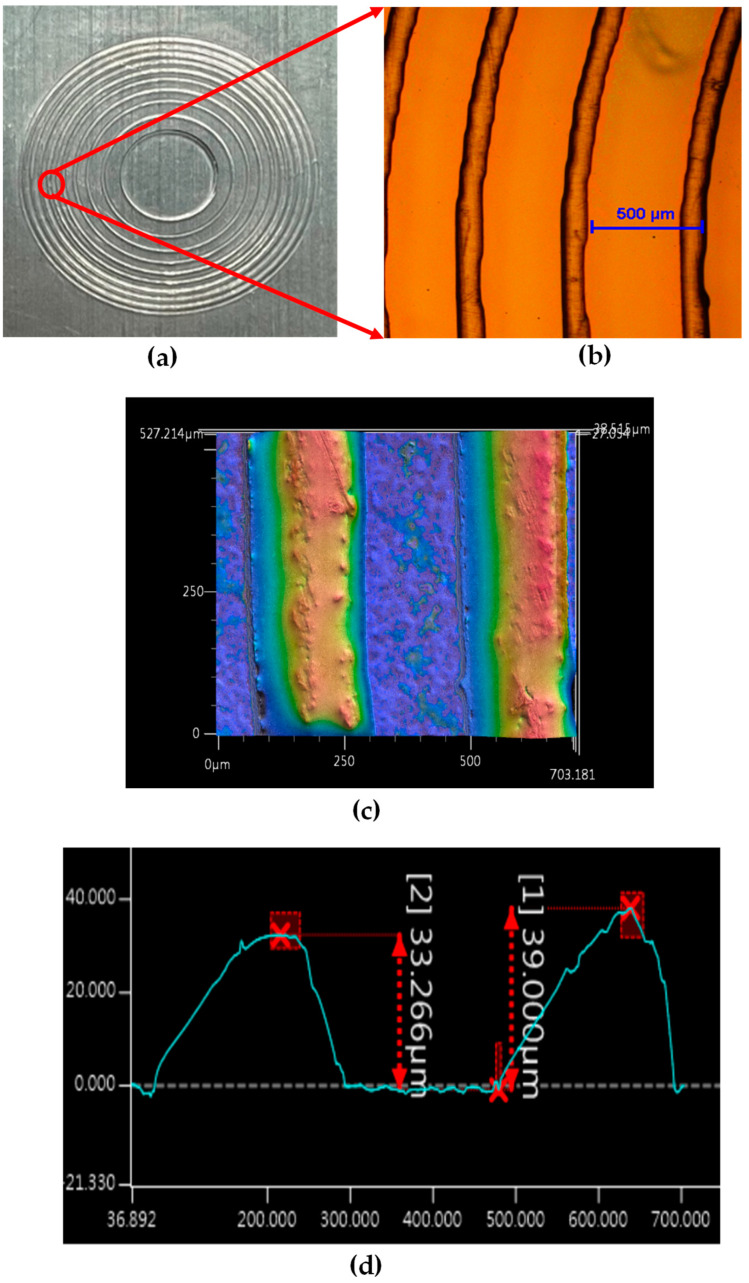
Measurements of the 3D-printed Fresnel zone plate structure: (**a**) device, (**b**) microscopic view, (**c**) stereoscopic profile image, and (**d**) structure depth measured with the stereoscopic profile meter.

The printed 3D structure on the ITO substrate was measured using an optical microscope (a Nikon microscope) and a stereoscopic profile meter (a Keyence 3D optical profiler) to ensure that the dimensions of the produced elements were the same as those in the design, as shown in Figure 9 and Figure 10.

**Figure 11:**
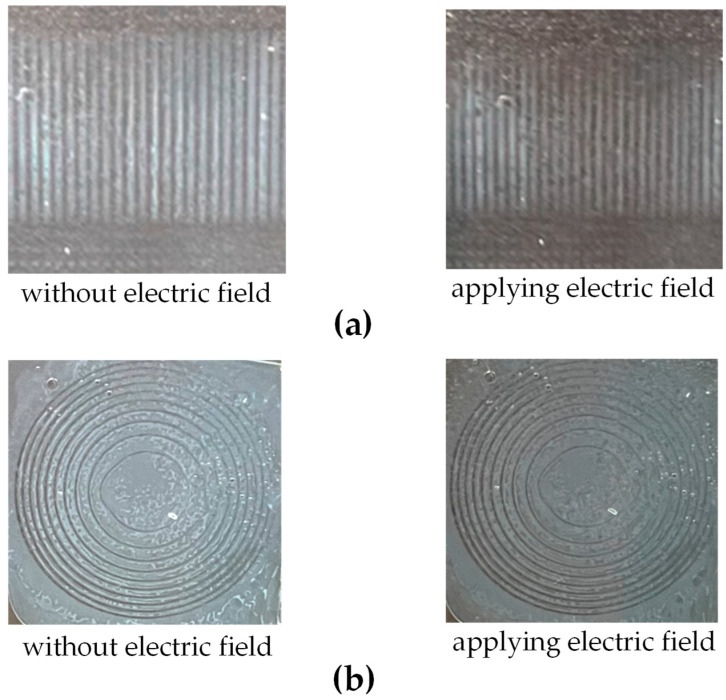
Pictures of the produced tunable components of (**a**) the diffraction grating and (**b**) the Fresnel zone plate when an electric field is (right side) and is not (left side) applied.

Next, an optimal mixture of LCs (~59 wt%, type: E7, index of refraction: $n_{e}=1.7472$, $n_{o}=1.5217$, ∆n=0.2255), transparent UV-curable resin (~40 wt%, Aqua-Clear; index of refraction: 1.512), and interface surfactant (~1 wt%, Span-80; index of refraction: 1.473) was uniformly generated by stirring to finish preparing the PDLC. Then, the PDLCs were dripped onto the printed structure on the ITO substrate and the other ITO substrate with the conducting surface was added to cover the structure/PDLC combination and sealed to form the bonded sandwich element. Finally, the assembly was post-cured inside a secondary UV curing chamber for 10 s to complete the process. Pictures of the final tunable diffraction grating and Fresnel zone plate when an electric field is and is not applied are shown in Figure 11. The photos on the left side of Figure 11a,b were taken by a camera when no electric field was applied. They exhibit higher contrast between adjacent structures with and without PDLCs, where the PDLCs scattered incident light to present bright images. For comparison, the photos on the left side of Figure 11a,b exhibit lower contrast and darker images, since the PDLCs aligned well and did not scatter light after applying an electric field. Although the structure was less obvious, it still did not completely disappear. This may be the reason the LC droplets of the PDLC were not fully oriented to match the index of refraction of the 3D-printed structure.

## 3. Experiments and Results

Based on the above implemented components, two experiments carried out for the purposes of measurement and verification were conducted as follows.

### 3.1. Diffraction Grating

**Figure 12:**
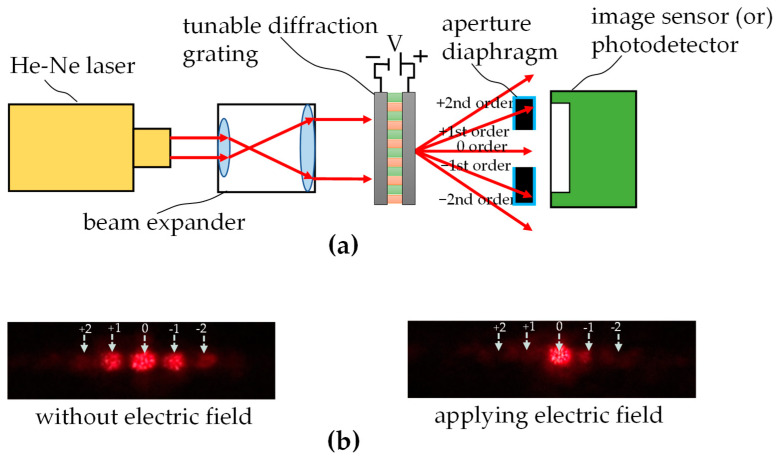
Experiments for verifying the diffraction grating: (**a**) experimental setup, (**b**) diffraction spot images when an electric field is and is not applied.

**Figure 13:**
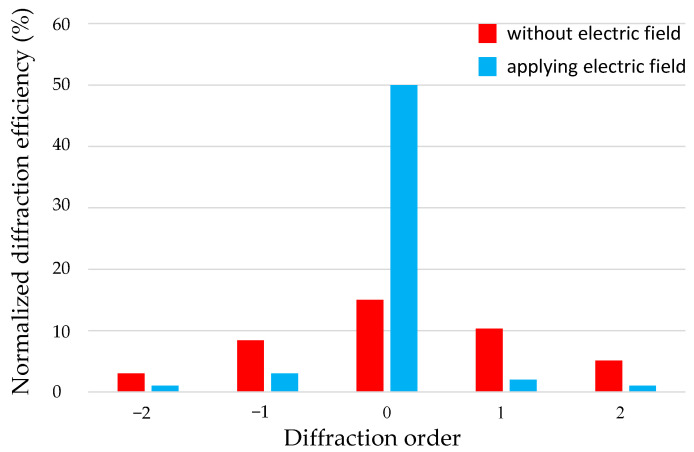
Diffraction efficiency measured for primary diffraction orders when an electric field is (applied voltage: 100 V) and is not applied.

Figure 12a depicts the experimental setup for investigating how the diffraction orders are influenced by applying an electric field onto the implemented diffraction grating. A He-Ne laser with a 632.8 nm wavelength was used as the light source, and a beam expander was used to expand the collimated laser beam diameter. The laser beam was incident on the device, and diffraction occurred. An image sensor was used to watch the change in primary diffraction orders corresponding to changes in the electric fields, as shown in Figure 12b. Furthermore, the image sensor was replaced with a photodetector to measure the diffraction efficiency of each diffraction order. The diffraction efficiency was calculated according to Equation (3), and the data are shown in Figure 13. From the data, we find that the tunable diffraction grating can change the intensities of the 0th, +1st, −1st, +2nd, and −2nd diffraction orders from 15%, 10%, 8%, 5%, and 3% before applying an electric field to 50%, 2%, 3%, 1%, and 1% after applying an electric field with a voltage of 100 V, respectively. The tunable diffraction grating significantly increases the efficiency of the 0th order by 35% and apparently suppresses that of high orders when an electric field is applied. The experiments show the expected results and demonstrate the ability of the proposed tunable diffraction grating to switch light efficiencies.

(3) $$Normalized\;diffraction\;efficiency=\frac{intensity\;of\;measured\;diffraction\;spot\;}{intensity\;of\;incident\;light\;on\;device}\times 100\%$$

### 3.2. Fresnel Zone Plate

**Figure 14:**
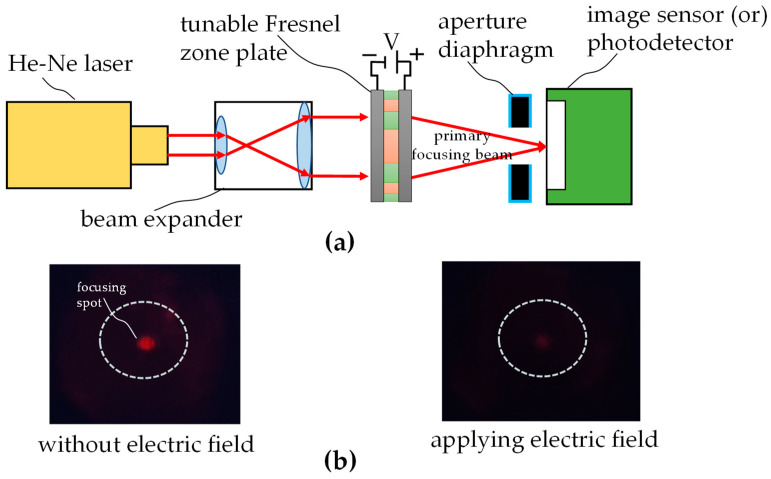
Experiments for verifying the Fresnel zone plate: (**a**) experimental setup, (**b**) focusing spot images when an electric field is and is not applied.

**Figure 15:**
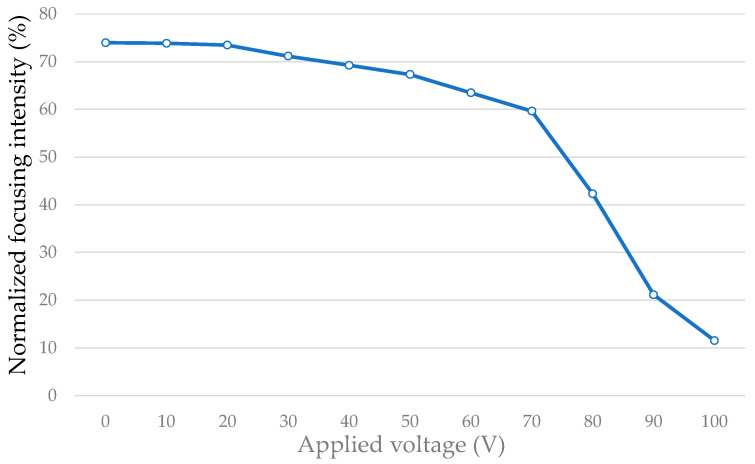
Focusing intensity measured on the focal plane corresponding to various applied voltages.

Figure 14a depicts the experimental setup for investigating how the focusing capability is influenced by applying an electric field onto the implemented Fresnel zone plate. The result is similar to that for the diffraction grating. The laser beam is incident on the element and focused by the element on the nominal focal plane. An image sensor is placed on the focal plane and used to watch the change in focusing spots corresponding to the variations in the electric field, as shown in Figure 14b. Furthermore, the image sensor is replaced with a photodetector to measure the focusing spot intensity. The focusing intensity is calculated according to Equation (4), and the data are shown in Figure 15 and Table 4. Transmittance of the element is around 74% when no electric field is applied. It starts from above 70 V for a more obvious response and decreases to a transmittance of around 12% at a saturated voltage of 100 V. The experiments also show expected results and demonstrate that the proposed tunable Fresnel zone plate has focusing capabilities.

(4) $$Normalized\;focusing\;intensity=\frac{intensity\;of\;measured\;focusing\;spot\;}{intensity\;of\;incident\;light\;on\;device}\times 100\%$$

## 4. Discussion and Conclusions

This study demonstrates the feasibility of combining polymer-dispersed liquid crystals with photopolymerization 3D printing to fabricate tunable micro-optical components. They were designed and manufactured, and various experiments were completed. They achieved the expected results and presented optical switchable and tunable properties that differed from those of conventional 3D-printed elements. Moreover, the proposed configuration creates an innovative method that simplifies the manufacturing processes for the conventional microfabrication of optical components.

The experimental results show that the implemented diffraction grating can provide obvious diffraction efficiency changes for primary orders, whereas the implemented Fresnel zone plate can provide variations in focusing light intensity. Nevertheless, there are still many challenges to overcome. First, due to the resolution limit of 3D printers, it is difficult for the structure spacing to be less than 100 μm, which restricts the diffraction angle and focal length for diffraction gratings and Fresnel zone plates, respectively. Second, the depth of each printing layer cannot be easily accurately controlled, which affects the actual light efficiency, which is apparently different from that of the simulation. The above two issues can be solved by looking for a 3D printer with higher resolution and thinner layer thickness. Third, because the turning voltage of PDLCs is above 70 V, additional circuits are needed to boost the ordinary low-voltage power supply. In order to reduce the voltage, the limitations related to the minimum thickness of a printed layer and the structural depth of a component must be improved, thereby reducing the PDLC thickness. Finally, although upper and lower glass ITO substrates are used to support the 3D-printed structure, photopolymerization with UV curing can sometimes lead to reduced mechanical rigidity, potentially distorting the geometry or dimensions of printed objects and therefore altering the optical performance of devices. This result has not yet been investigated in this study and may need to be verified. All the above require further research and will be explored in future work.

## Figures and Tables

**Table 1:** Parameters for the diffraction grating simulation using Gsolver.

Parameters	Wavelength (nm)	Incident Angle (°)	Index of Refraction (Polymer)	Index of Refraction (Outside)	Grating Period (μm)	Duty Cycle
Value	632.8	0	1.5	1	500	25%

**Table 2:** Calculated radii of ten zones for the targeted zone plate with a focal length of 8 m.

Zone Radius	$r_{1}$	$r_{2}$	$r_{3}$	$r_{4}$	$r_{5}$	$r_{6}$	$r_{7}$	$r_{8}$	$r_{9}$	$r_{10}$
Value (mm)	2.249	3.181	3.897	4.499	5.031	5.511	5.951	6.364	6.749	7.115

**Table 3:** Main parameters for the preprocessing software setting.

Parameter	Structure Resolution	Layer Thickness	Exposure Time	Pull Speed
Value	22 μm	50 μm	13 sec.	10 mm/min.

**Table 4:** Measured data for focusing spot when an electric field is and is not applied.

Applied voltage (V)	0	10	20	30	40	50
Focusing intensity	74%	74%	74%	71%	69%	67%
Applied voltage (V)	60	70	80	90	100	
Focusing intensity	63%	60%	42%	21%	12%	

## Data Availability

The data presented in this article are not currently publicly available but are available from the authors on reasonable request.
